# *PUNISHER* rs12318065 C>A transversion: a putative somatic driver mutation for poor prognosis in colon cancer

**DOI:** 10.1042/BSR20220465

**Published:** 2022-06-27

**Authors:** Sameerah Shaheen, Eida M. Alshammari, Sara H. Mokhtar, Aliah R. Alshanwani, Eman A. Toraih, Afaf T. Ibrahiem, Manal S. Fawzy, Shymaa Ahmed Maher

**Affiliations:** 1 Anatomy Department and Stem Cell Unit, College of Medicine, King Saud University, Riyadh, Saudi Arabia; 2 Department of Chemistry, College of Science, University of Ha’il, Ha’il, Saudi Arabia; 3 Medical Laboratory Technology Department, Faculty of Applied Medical Sciences, King Abdulaziz University, Jeddah, Saudi Arabia; 4 Physiology Department, College of Medicine, King Saud University, Riyadh, Saudi Arabia; 5 Division of Endocrine and Oncologic Surgery, Department of Surgery, Tulane University, School of Medicine, New Orleans, Louisiana, U.S.A.; 6 Genetics Unit, Department of Histology and Cell Biology, Faculty of Medicine, Suez Canal University, Ismailia, Egypt; 7 Department of Pathology, Faculty of Medicine, Northern Border University, Arar, Saudi Arabia; 8 Department of Pathology, Faculty of Medicine, Mansoura University, Mansoura, Egypt; 9 Department of Biochemistry, Faculty of Medicine, Northern Border University, Arar, Saudi Arabia; 10 Department of Medical Biochemistry and Molecular Biology, Faculty of Medicine, Suez Canal University, Ismailia, Egypt; 11 Center of Excellence in Molecular and Cellular Medicine (CEMCM), Faculty of Medicine, Suez Canal University, Ismailia, Egypt

**Keywords:** colorectal cancer, Prognosis, PUNISHER, rs12318065, single nucleotide polymorphisms, survival

## Abstract

Objective: Colon cancer (CC) remains one of the leading causes of cancer death worldwide. Several mutations/polymorphisms have been implicated in CC development and/or progression. The role of the recently identified variants related to the long non-coding RNAs (lncRNAs) family has not yet been fully uncovered. In this sense, we aimed to explore the association between the lncRNA *PUNISHER* rs12318065 variant and the CC risk and/or prognosis. Methods: A total of 408 CC (paired 204 cancer/non-cancer) tissues were genotyped using the TaqMan allelic discrimination assay. Results: “A” variant was associated with higher susceptibility to develop CC under heterozygote (A/C vs. C/C: OR = 1.39, 95%CI = 1.09–2.17, *P*=0.002), homozygote (A/A vs. C/C: OR = 2.63, 95%CI = 1.51–4.58, *P*=0.001), dominant (A/C-A/A vs. C/C: OR = 1.72, 95%CI = 1.15–02.57, *P*=0.008), and recessive (A/A vs. C/C-A/C: OR = 2.23, 95%CI = 1.34–3.72, *P*=0.001) models. Patients with metastasis were more likely to harbor A/A and A/C genotypes (16.7% and 14.1%) than 11% with the C/C genotype (*P*=0.027). Patients harboring C>A somatic mutation were more likely to develop relapse (52.6% vs. 26.5%, *P*=0.003), have poor survival (57.9% vs. 27.7%, *P*=0.001), and have shorter disease-free survival (43.2 ± 2.6 months vs. 56.8 ± 1.29 months, *P*<0.001) and overall survival (49.6 ± 2.4 months vs. 56.6 ± 0.99 months, *P*<0.001). Multivariate Cox regression analysis showed that patients with distal metastasis and C>A somatic mutation were three times more likely to die. Conclusions: To our knowledge, the present study is the first to identify that the *PUNISHER* rs12318065 variant could be a novel putative driver of colon cancer and is associated with poor prognosis.

## Introduction

Colon cancer (CC) is a heterogeneous disease that represents one of the most common (1.9 million incidence rate) and leading causes of cancer-related mortality (0.9 million death in 2020) worldwide [1]. In addition to increased exposure to environmental risk factors due to shifting diet and lifestyle toward westernization, genetic elements have also been reported to contribute to increasing CC incidence [2,3]. Despite current advances in surgical therapy, chemotherapy, and molecular targeting therapy for CC, the overall 5-year survival rate for patients remains as low as 12% for metastatic cases of CC [4]. Therefore, understanding the molecular mechanisms involved in CC development, progression, and metastasis is critical for developing specific diagnostic methods and individualized therapeutic strategies [5].

Recently, long non-coding RNAs (lncRNAs) have been identified as new crucial regulators of diverse cellular processes, including cell proliferation, differentiation, and cancer cell metastasis [6]. Accumulating evidence has revealed that aberrant lncRNA expression plays an essential role in carcinogenesis and tumor progression [7,8]. Interestingly, these lncRNAs are involved in modulating an extensive range of cellular processes, including reprogramming stem cell pluripotency, parental imprinting, and cancer cell proliferation and metastasis through chromatin remodeling, epigenetic modification, and miRNA sponging [9].

The oncogenic lncRNA PUNISHER, also known as “AGAP2-AS1 (ADP-ribosylation factor [Arf], GTPase-activated protein [GAP], isoform 2-antisense RNA 1)”, was recently reported to augment cell viability and mobility and confers gemcitabine resistance by inhibiting microRNA-497 in colorectal cancer (CRC) [10]. It has been found that PUNISHER could promote CRC cell proliferation, migration, and epithelial-to-mesenchymal transition, inhibit apoptosis, and enhance the chemoresistance of CRC cells to gemcitabine [10,11].

Although *PUNISHER* gene expression has been explored in several cancers, including CRC [10–19]; however, the mutation pattern and the biological impact of the related gene variant(s) in CC remain largely unknown.

Several studies have reported the relationship between lncRNA gene variants and colon cancer risk and/or prognosis [20]. For example, Xu et al. demonstrated that patients with rs7958904 CC genotype of “HOX transcript antisense RNA” *HOTAIR* had decreased risk of CRC [21]. Similarly, Zheng et al. explored the association of the G allele of rs2288947 of the “tissue differentiation-inducing non-protein coding RNA” (*TINCR*) with a 23% decreased CRC risk. In comparison, the A allele of rs8105637 for the latter lncRNA was significantly associated with a 22% increased risk of CRC, and both variants were associated with lymph node metastasis occurrence [22]. Zhu et al. were the first to report that the functional indel rs145204276 variant within the promoter of the “growth arrest-specific 5” (*GAS5*) could modulate CRC risk by impacting the gene transcription activity [23]. Also, Li et al. found that “carriers of rs2839698 A allele for lncRNA *H19* had a significantly increased risk of CRC, compared to those carrying G allele” [24]. Collectively, these reports confirmed the potential association of several lncRNA variants with CRC risk and/or prognosis.

The rs12318065 C>A polymorphism is an *AGAP2* 3`-UTR variant, and an *AGAP2-AS1* (i.e. *PUNISHER*) intronic variant located at chromosome 12:57726493 according to the “Genome Reference Consortium Human Build 38 patch release 13 (GRCh38.p13)” (https://www.ncbi.nlm.nih.gov/snp/?term=rs12318065) (last accessed February 22, 2022). Based on searching the *PUNISHER* gene variants in the dbSNP (www.ncbi.nlm.nih.gov) for a minor allele frequency (MAF) ≥ 0.1 and the absence of previous studies exploring the impact of this variant on CC risk and/or outcome, we were interested in performing allelic discrimination analysis of paired CC and non-cancer tissues as a preliminary step for future related full-scale variant studies. The recognition of the selected variant association with CC risk and/or prognosis may be helpful with other genetic/epigenetic and environmental markers to develop prognostic models for CC targeted management in the near future.

## Materials and methods

### Study population

A total of 408 retrospectively collected tissue specimens were analyzed in the present study population (204 cancer tissues were compared with their corresponding non-cancer adjacent tissues). The “formalin-fixed, paraffin-embedded (FFPE)” samples were archived in the “Suez Canal University hospital-pathology lab, Ismailia, and the Oncology Center of Mansoura Hospital, Mansoura, Egypt, from January 2008 to December 2018. The inclusion criteria included archived paired primary colon cancer tissue samples with no history of preoperative radiotherapy/chemotherapy treatment with the availability of the related clinicopathological patients’ characteristics from the medical records, including the follow-up survival data. The cancer staging system was according to the “International Union Against Cancer TNM staging system (8^th^ ed.)” [25]. The exclusion criteria included samples with incomplete (clinical and/or follow-up) data, unavailability of paired non-cancer tissues, history of receiving any preoperative therapeutic regimen, secondary CC, insufficient tissue samples, non-homogenous or histologically well-characterized samples, or inadequate quantity/quality of tissue sample-extracted DNA. “Declaration of Helsinki” guidelines were followed, and the “Suez Canal University-Faculty of Medicine-Medical Research Ethics Committee” approved the present study. Patient consent was waived as the included samples of this retrospective study were archived.

### Histopathological assessment

**Figure 1:**
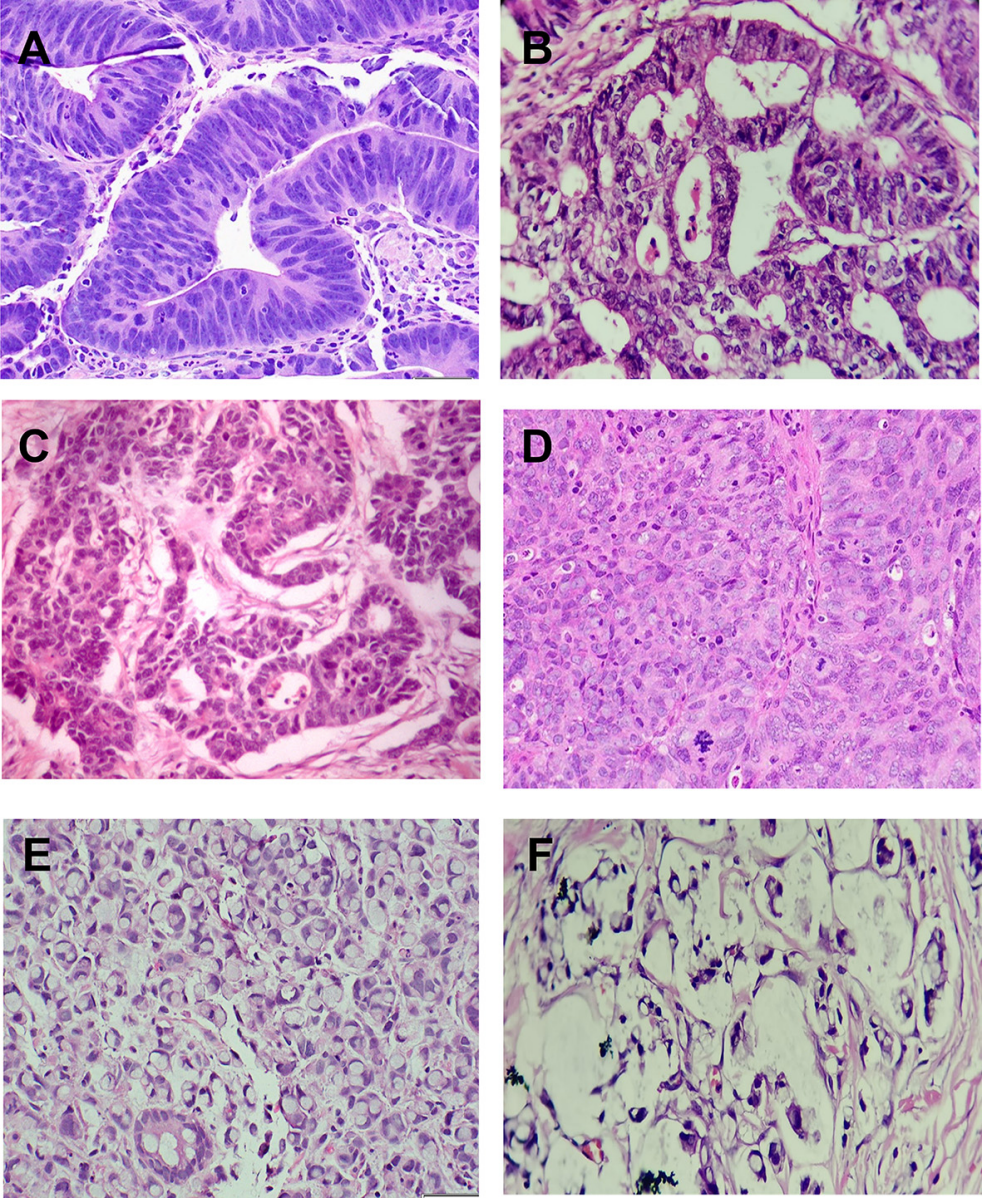
Hematoxylin and eosin (H&E) staining of colon cancer specimens Panel (**A**) showed colonic tubular adenoma with a high degree of dysplasia (×200). Panel (**B**) showed moderately differentiated colon adenocarcinoma formed of more than 50% of invasive irregular separate glands (×200). Panel (**C**) showed poorly differentiated colon adenocarcinoma showed sheets and irregular fused glands infiltrating the wall (×200). Panel (**D**) showed undifferentiated colon adenocarcinoma showed diffuse sheets of anaplastic cells (×400). Panel (**E**) showed intramucosal signet ring carcinoma (×200), and panel (**F**) showed mucinous colonic carcinoma that showed lakes and pools with mucin with floating malignant cells and fragments of acini (×200).

**Figure 2:**
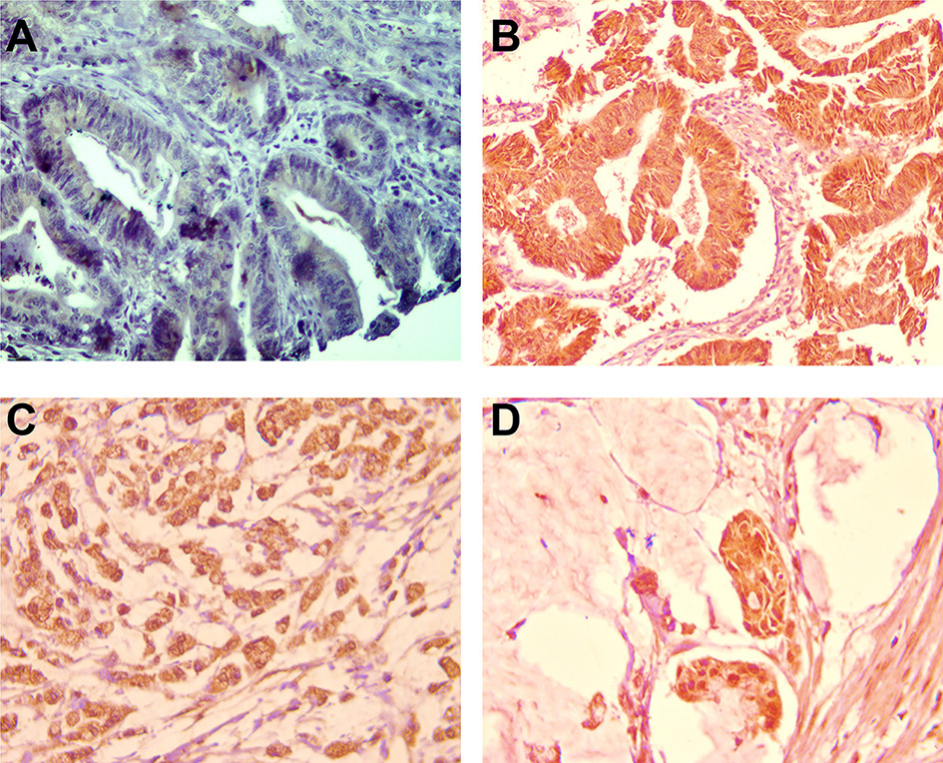
Immunohistochemical (IHC) staining of colon carcinoma specimens for BRAF (**A**) Negative staining for BRAF monoclonal antibody in colonic tubular adenoma (×200). (**B**) Diffuse cytoplasmic staining for BRAF monoclonal antibody in well-differentiated colon adenocarcinoma (×200). (**C**) Diffuse cytoplasmic staining for BRAF monoclonal antibody in tumor cells of signet ring carcinoma of the colon (×200), and (**D**) diffuse cytoplasmic staining in the floating malignant epithelial cells of colonic mucinous carcinoma (×200).

The present samples included 138 adenocarcinomas (67.6%), 30 mucinous (14.7%), 26 signet ring cells (12.7%), and 10 undifferentiated (4.9%) carcinoma cases. For all samples, the original hematoxylin and eosin (H&E)-stained sections were re-investigated to confirm the histopathological diagnosis (Figure 1) and record the histopathological parameters as “histological type, grading/staging of cancer, tumor invasion in the wall, circumferential margin, lymph node metastasis (LNM), and lymphovascular invasion (LVI)”. Five-micrometer thick tissue sections were prepared and examined for BRAF analysis according to Rashid et al. [26] (Figure 2). All the quality control measurements were applied accordingly.

### Allelic discrimination analysis

Tissue DNA was isolated from samples via “QIAamp DNA FFPE Tissue Kit” (Catalog No. 56404, Qiagen, Hilden, Germany). After dissolving and removing the paraffin by xylene, the samples were lysed under denaturing conditions with proteinase K, and the genomic DNA was obtained following the manufacturer’s protocol. NanoDrop ND-1000 (NanoDrop Technologies, Inc. Wilmington, DE, U.S.A.) was used to assess the concentration/purity of the extracted DNA. A specific TaqMan probe-fluorescence assay (C__30952613_10) with VIC and FAM dyes “GAGTGGGTGCGTCTGTCCAGCGGTC[A/C]GCCCGGTGTGGTCGTGCCCGGCCCG” for each allele, respectively, was run for allelic discrimination. Real-Time polymerase chain reaction (PCR) was carried out by two coauthors independently blinded to cancer/non-cancer sample status in a StepOne™ Real-Time PCR System (Applied Biosystems, Foster City, CA, U.S.A.) as previously described [27]. Loading of DNase-free water instead of unknown DNA in each run as a negative control was applied. The PCR reaction conditions were run as follows: “40 cycles at 95°C for 10 min, 95°C for 15 s, annealing at 60°C for 1 min, and final step at 60°C for 30 s” [28]. Five percent of the samples were randomly selected and run in duplicates to ensure results reliability with a 100% concordance rate for genotype calls. The genotyping results were retrieved by the related SDS software version 1.3.1.

### Statistical analysis

Data analysis was performed using the “Statistical Package for the Social Sciences (SPSS) for Windows” software (version 27.0). Genotype/allele frequencies and Hardy–Weinberg equilibrium analysis were performed using the online SNPStats software (https://www.snpstats.net/). Binary logistic regression was performed, and adjusted odds ratio (OR) and 95% confidence interval (CI) by age and sex were estimated for five genetic association models (homozygote and heterozygote comparison, dominant, recessive, and over-dominant models). McNemar’s test was used to calculate the somatic mutation rate [29]. Categorical variables were presented as frequencies and percentages and compared using the chi-square (*χ*2) or Fisher’s exact tests when appropriate. Continuous variables were shown as mean ± standard deviation and compared using the Student’s *t*-test. A two-tailed *P*-value of <0.05 was considered statistically significant. Cox Proportional Hazards regression analysis was applied to detect predictors of poor survival. Kaplan–Meier survival curves were generated to compare patients with and without C to A somatic mutation.

## Results

### Characteristics of the study population

The study included paired samples of 204 colon cancer patients. The mean age was 58.3 years ± 12.3, and 60.8% were men. Of these, 68 patients (33.3%) died during the follow-up period of over five years. Those who expired were more likely to be men (70.6% vs. 55.9%, *P*=0.049), have lesions in the transverse or descending colon (57.4% vs. 45.1%, *P*=0.001), presented with poorly differentiated pathological grade (42.6% vs. 20.6%, *P*=0.003), advanced lymph node metastasis (29.9% vs. 10.4%, *P*<0.001), and distal metastasis (27.9% vs. 11%, *P*=0.005) (Table 1).

**Table 1:** Baseline characteristics of the study population

Variable	Total (*n*=204)	Survived (*n*=136)	Died (*n*=68)	*P*-value
**Age (y)**				
**≤60**	106 (52)	68 (50)	38 (55.9)	0.46
**>60**	98 (48)	68 (50)	30 (44.1)	
**Sex**				
**Men**	124 (60.8)	76 (55.9)	48 (70.6)	**0.049**
**Women**	80 (39.2)	60 (44.1)	20 (29.4)	
**Location**				
**Right**	112 (54.9)	83 (61)	29 (42.6)	**0.001**
**Transverse**	10 (4.9)	2 (1.5)	8 (11.8)	
**Left**	82 (40.2)	51 (37.5)	31 (45.6)	
**Type**				
**Adenocarcinoma**	138 (67.6)	90 (66.2)	48 (70.6)	**0.033**
**Mucinous**	30 (14.7)	23 (16.9)	7 (10.3)	
**Signet cell**	26 (12.7)	20 (14.7)	6 (8.8)	
**Undifferentiated**	10 (4.9)	3 (2.2)	7 (10.3)	
**Grade**				
**G1**	24 (11.8)	16 (11.8)	8 (11.8)	**0.003**
**G2**	123 (60.3)	92 (67.6)	31 (45.6)	
**G3**	57 (27.9)	28 (20.6)	29 (42.6)	
**T stage**				
**T1**	23 (11.3)	14 (10.3)	9 (13.2)	0.12
**T2**	99 (48.5)	67 (49.3)	32 (47.1)	
**T3**	51 (25)	39 (28.7)	12 (17.6)	
**T4**	31 (15.2)	16 (11.8)	15 (22.1)	
**N stage**				
**N0**	82 (40.2)	49 (36)	33 (48.5)	**<0.001**
**N1**	88 (43.1)	73 (53.7)	15 (22.1)	
**N2**	34 (16.7)	14 (10.3)	20 (29.4)	
**M stage**				
**M0**	170 (83.3)	121 (89)	49 (72.1)	**0.005**
**M1**	34 (16.7)	15 (11)	19 (27.9)	
**Lymphovascular invasion**				
**No**	135 (66.2)	92 (67.6)	43 (63.2)	0.53
**Yes**	69 (33.8)	44 (32.4)	25 (36.8)	
**Duke’s stage**				
**A**	56 (27.5)	36 (26.5)	20 (29.4)	**0.004**
**B**	22 (10.8)	13 (9.6)	9 (13.2)	
**C**	94 (46.1)	73 (53.7)	21 (30.9)	
**D**	32 (15.7)	14 (10.3)	18 (26.5)	
**BRAF mutation**				
**Wild-type**	144 (70.6)	98 (72.1)	46 (67.6)	0.51
**Mutant**	60 (29.4)	38 (27.9)	22 (32.4)	
**Relapse**				
**No**	140 (68.6)	98 (72.1)	42 (61.8)	0.15
**Yes**	64 (31.4)	38 (27.9)	26 (38.2)	

Data are presented as frequency (percentage). Two sided-Chi-square test was used. The bold values indicate statistical significance at a *P*-value below 0.05.

### Single-nucleotide polymorphism (SNP) analysis of *PUNISHER* variant

**Figure 3:**
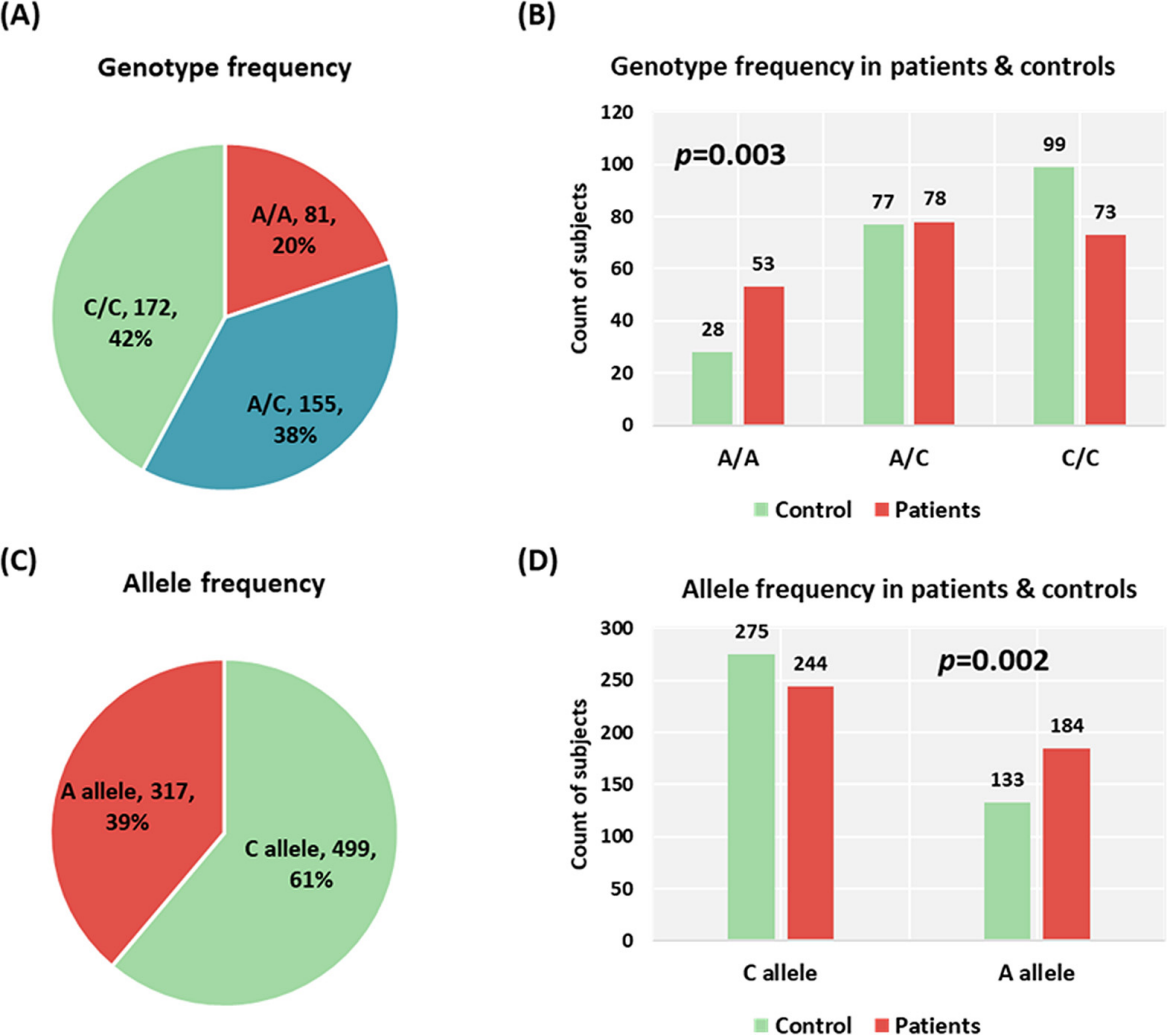
Genotype and allele frequencies of *PUNSHER* (AGAP2-AS1) rs12318065 variant Data are presented as frequency and percentage. A two-sided Chi-square test was used. Statistical analysis was set at a *P*-value below 0.05.

Genotype frequency of rs12318065 agreed with HWE in the control group (*P*=0.06). MAF (A allele) accounted for 0.33 in controls. According to the 1000 Genome Project, the same allele frequencies were 0.02 in Africans, 0.10 in Asians, 0.25 in Americans, and 0.13 in Europeans. In comparison between malignant and adjacent colon tissues, a higher frequency of the A allele was more representative in cancer tissues compared with control tissues (45% vs. 33%, *P*=0.002). Correspondingly, A/A and C/A genotypes were more prevalent in cancer specimens (26.0% and 38.2%) compared with counterpart non-cancer control tissues (13.7% and 37.7%, *P*=0.003) (Figure 3).

### Impact of genotypes on cancer risk

**Figure 4:**
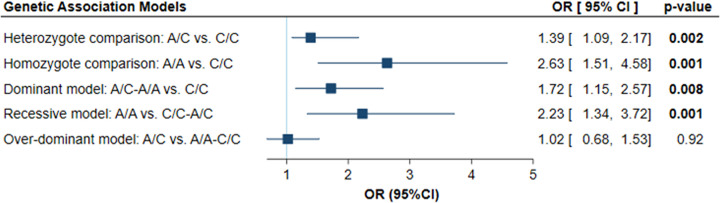
Genetic association models for PUNISHER gene variant and cancer risk Regression analysis was adjusted by the age and sex of the patients. Adjusted odds ratio and confidence interval are shown.

As depicted in Figure 4, a variant was associated with higher susceptibility to develop colon cancer under heterozygote comparison (A/C vs. C/C: OR = 1.39, 95%CI = 1.09–2.17, *P*=0.002), homozygote comparison (A/A vs. C/C: OR = 2.63, 95%CI = 1.51–4.58, *P*=0.001), dominant model (A/C-A/A vs. C/C: OR = 1.72, 95%CI = 1.15–2.57, *P*=0.008), and recessive model (A/A vs. C/C-A/C: OR = 2.23, 95%CI = 1.34–3.72, *P*=0.001).

### Somatic mutation burden analysis

Tumor-normal paired analysis revealed genotype concordance in 166 out of 204 tissue samples, accounting for 81.4% of patients. In contrast, 38 samples showed the allelic difference between paired samples with a higher representation of the A allele in tumor samples. The C/C and A/C genotypes in 13 and 12 patients, respectively, were mutated to the A/A genotype in paired (cancer and non-cancer) tissues. In addition, 13 other cases with C/C genotype showed a change in one gene locus to A/C (Table 2).

**Table 2:** Somatic mutations of rs12318065 (A/G) genotypes in cancer and paired non-cancer tissues

Genotypes	Cancer			*P*-value
	A/A	A/C	C/C	
**Control**				
**A/A**	28 (52.8)	0 (0)	0 (0)	<0.001
**A/C**	12 (22.6)	65 (83.3)	0 (0)	
**C/C**	13 (24.5)	13 (16.7)	73 (100)	

Values are shown as numbers (% of total participants). McNemar’s test was used. The bold value indicates statistical significance at a *P*-value below 0.05.

### Association of *PUNISHER* genotypes with clinical and pathological features

*PUNISHER* rs12318065 genotypes were associated with distal metastasis; patients with metastasis were more likely to harbor A/A and A/C genotypes (16.7% and 14.1%) compared with 11% with C/C genotype (*P*=0.027). In addition, a higher frequency of mortality was reported in A/A (33.3%) and A/C (32.1%) groups compared with the C/C genotype (23.3%) (Table 3). A comparison between tumor samples harboring switch to A variant compared with counterparts is shown in Table 4. Patients harboring C>A somatic mutation were more likely to develop relapse (52.6% vs. 26.5%, *P*=0.003) and have poor survival (57.9% vs. 27.7%, *P*=0.001).

**Table 3:** Association of *PUNISHER* genotypes with the clinical and pathological features

Variable	A/A (*n*=53)	A/C (*n*=78)	C/C (*n*=73)	*P*-value
**Age (years)**				
**≤60**	124 (60.8)	51 (65.4)	36 (49.3)	0.46
**>60**	80 (39.2)	27 (34.6)	37 (50.7)	
**Sex**				
**Women**	124 (60.8)	51 (65.4)	36 (49.3)	**0.038**
**Men**	80 (39.2)	27 (34.6)	37 (50.7)	
**Location**				
**Right**	112 (54.9)	46 (59)	39 (53.4)	0.12
**Transverse**	10 (4.9)	1 (1.3)	3 (4.1)	
**Left**	82 (40.2)	31 (39.7)	31 (42.5)	
**Type**				
**Adenocarcinoma**	138 (67.6)	54 (69.2)	52 (71.2)	0.61
**Mucinous**	30 (14.7)	10 (12.8)	8 (11.0)	
**Signet cell**	26 (12.7)	9 (11.5)	10 (13.7)	
**Undifferentiated**	10 (4.9)	5 (6.4)	3 (4.1)	
**Grade**				
**G1**	147 (72.1)	60 (76.9)	50 (68.5)	0.47
**G2/3**	57 (27.9)	18 (23.1)	23 (31.5)	
**T stage**				
**T1/2**	122 (59.8)	53 (67.9)	42 (57.5)	0.13
**T3/4**	82 (40.2)	25 (32.1)	31 (42.5)	
**Lymph node metastasis**				
**Negative**	82 (40.2)	31 (39.7)	30 (41.1)	0.98
**Positive**	122 (59.8)	47 (60.3)	43 (58.9)	
**Distal metastasis**				
**Negative**	170 (83.3)	67 (85.9)	65 (89)	**0.027**
**Positive**	34 (16.7)	11 (14.1)	8 (11.0)	
**Lymphovascular invasion**				
**Negative**	135 (66.2)	53 (67.9)	48 (65.8)	0.89
**Positive**	69 (33.8)	25 (32.1)	25 (34.2)	
**Duke’s stage**				
**A/B**	119 (58.3)	52 (66.7)	37 (50.7)	0.13
**C/D**	85 (41.7)	26 (33.3)	36 (49.3)	
**BRAF mutation**				
**Wild-type**	144 (70.6)	55 (70.5)	52 (71.2)	0.98
**Mutant**	60 (29.4)	23 (29.5)	21 (28.8)	
**Relapse**				
**Negative**	140 (68.6)	57 (73.1)	52 (71.2)	0.17
**Positive**	64 (31.4)	21 (26.9)	21 (28.8)	
**Mortality**				
**Negative**	136 (66.7)	53 (67.9)	56 (76.7)	**0.010**
**Positive**	68 (33.3)	25 (32.1)	17 (23.3)	
**DFS (Months)**				
**Prolonged (≥48)**	64 (31.4)	26 (33.3)	25 (34.2)	0.45
**Short (<48)**	140 (68.6)	52 (66.7)	48 (65.8)	
**OS (Months)**				
**Prolonged (≥48)**	94 (46.1)	38 (48.7)	40 (54.8)	**0.020**
**Short (<48)**	110 (53.9)	40 (51.3)	33 (45.2)	

Data are presented as frequency (percentage). Two sided-Chi-square test was used. The bold values indicate statistical significance at a *P*-value below 0.05. *n*: number; DFS: disease-free survival; OS: overall survival.

**Table 4:** Comparison between the somatic mutation and tumor phenotype

Variable	Without C>A mutation (*n*=166)	With C>A mutation (*n*=38)	*P*-value	OR (95%CI)
**Age (y)**				
**≤60**	88 (53)	18 (47.4)	0.59	*Reference*
**>60**	78 (47)	20 (52.6)		1.25 (0.62–2.54)
**Sex**				
**Female**	99 (59.6)	25 (65.8)	0.58	*Reference*
**Male**	67 (40.4)	13 (34.2)		0.77 (0.37–1.61)
**Location**				
**Right**	93 (56)	19 (50)	0.57	*Reference*
**Transverse**	7 (4.2)	3 (7.9)		2.09 (0.49–8.85)
**Left**	66 (39.8)	16 (42.1)		1.18 (0.56–2.47)
**Type**				
**Adenocarcinoma**	114 (68.7)	24 (63.2)	0.65	*Reference*
**Mucinous**	22 (13.3)	8 (21.1)		1.72 (0.68–4.33)
**Signet cell**	22 (13.3)	4 (10.5)		0.86 (0.27–2.73)
**Undifferentiated**	8 (4.8)	2 (5.3)		1.18 (0.23–5.94)
**Grade**				
**G1**	121 (72.9)	26 (68.4)	0.55	*Reference*
**G2/3**	45 (27.1)	12 (31.6)		1.24 (0.58–2.67)
**T stage**				
**T1/2**	102 (61.4)	20 (52.6)	0.36	*Reference*
**T3/4**	64 (38.6)	18 (47.4)		1.43 (0.71–2.92)
**Lymph node metastasis**				
**Negative**	71 (42.8)	11 (28.9)	0.14	*Reference*
**Positive**	95 (57.2)	27 (71.1)		1.83 (0.85–3.94)
**Distal metastasis**				
**Negative**	140 (84.3)	30 (78.9)	0.46	*Reference*
**Positive**	26 (15.7)	8 (21.1)		1.44 (0.59–3.48)
**Lymphovascular invasion**				
**Negative**	105 (63.3)	30 (78.9)	0.08	*Reference*
**Positive**	61 (36.7)	8 (21.1)		0.46 (0.2–1.06)
**Duke's stage**				
**A/B**	96 (57.8)	23 (60.5)	0.85	*Reference*
**C/D**	70 (42.2)	15 (39.5)		0.89 (0.44–1.84)
**BRAF mutation**				
**Wild type**	118 (71.1)	26 (68.4)	0.84	*Reference*
**Mutant**	48 (28.9)	12 (31.6)		1.13 (0.53–2.43)
**Relapse**				
**No**	122 (73.5)	18 (47.4)	**0.003**	*Reference*
**Yes**	44 (26.5)	20 (52.6)		3.08 (1.49–6.36)
**Died**				
**No**	120 (72.3)	16 (42.1)	**0.001**	*Reference*
**Yes**	46 (27.7)	22 (57.9)		3.59 (1.73–7.43)
**DFS (months)**				
**Prolonged (≥48)**	56 (33.7)	8 (21.1)	0.17	*Reference*
**Short (<48)**	110 (66.3)	30 (78.9)		1.91 (0.82–4.44)
**OS (months)**				
**Prolonged (≥48)**	84 (50.6)	10 (26.3)	**0.007**	*Reference*
**Short (<48)**	82 (49.4)	28 (73.7)		2.87 (1.31–6.28)

Data are presented as frequency (percentage). *N*: number; DFS: disease-free survival; OS: overall survival. A two sided-Chi-square test was used. Binary logistic regression analysis was performed. Odds ratio (OR) and 95% confidence intervals (CI) are shown. The bold values indicate statistical significance at a *P*-value below 0.05.

### Survival analysis

**Figure 5:**
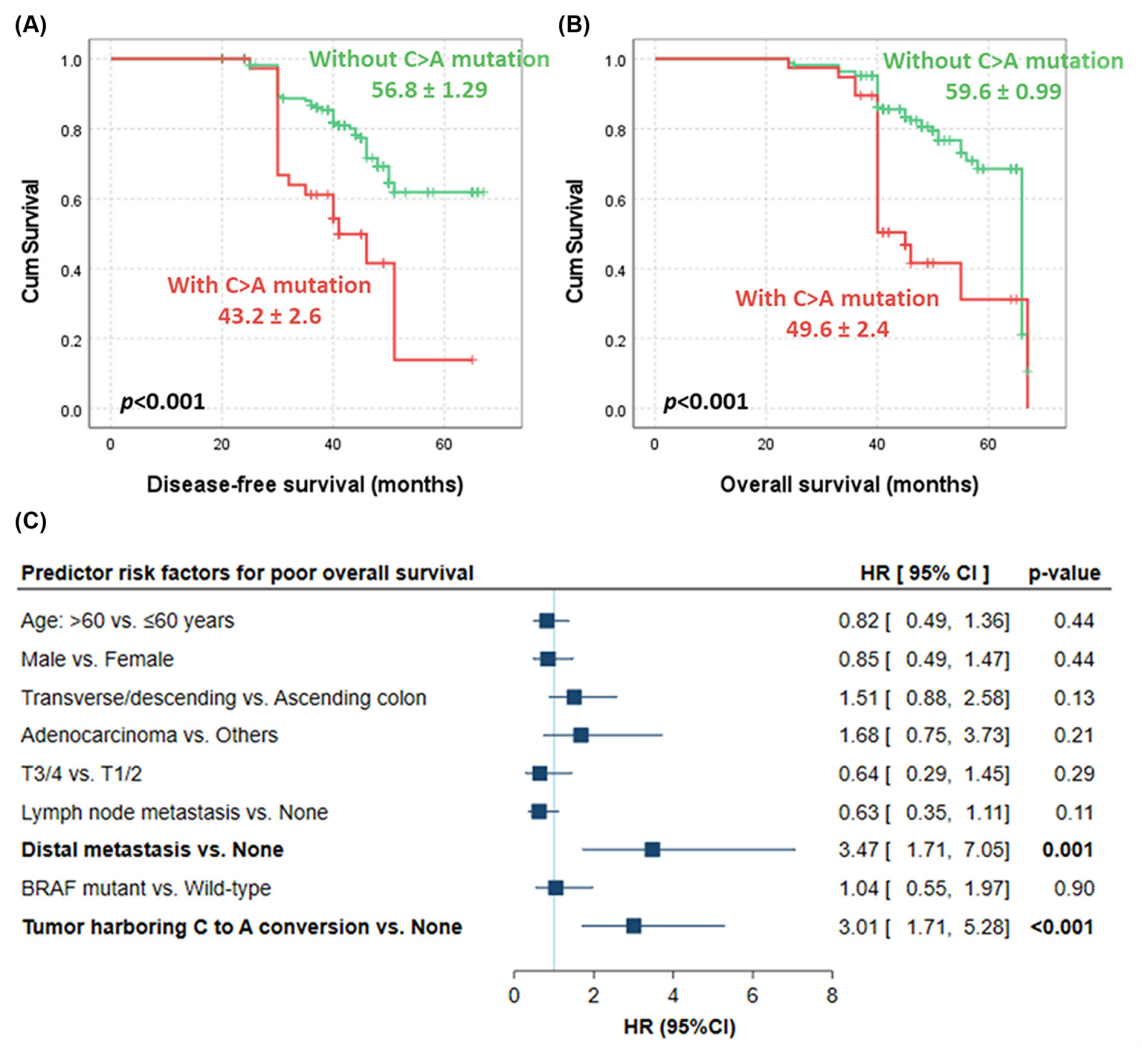
Survival analysis in patients with colon cancer (**A** and **B**) Kaplan–Meier survival curves illustrating the differential effect of *PUNISHER* somatic mutation on disease-free survival times. Survival times are shown as means and standard errors. Log Rank test was used to compare the difference between the groups. (**C**) Independent predictor risk factors for overall survival. Cox Proportional Hazard Regression analysis was performed. Hazard ratio and confidence interval are shown.

In comparison between patients who had C-to-A somatic mutation and non-cancer counterparts, Kaplan–Meier curves showed patients harboring conversion had shorter disease-free survival (43.2 ± 2.6 months vs. 56.8 ± 1.29 months, *P*<0.001) and overall survival times (49.6 ± 2.4 months vs. 56.6 ± 0.99 months, *P*<0.001). Multivariate Cox regression analysis showed distal metastasis (HR = 3.47, 95%CI = 1.71–7.05, *P*=0.001) and C-to-A somatic mutation (HR = 3.01, 95%CI = 1.71–5.28, *P*<0.001) were three times more likely to die (Figure 5).

## Discussion

Accumulating evidence recognized the impact of gene polymorphism in increased colon cancer risk through known and yet unknown cellular and molecular changes [30]. A slew of research has discovered that lncRNA SNPs are substantially associated with cancer risk [31]. The lncRNA gene-related polymorphisms may have a significant impact on lncRNA expression levels, processing, or secondary structure, culminating in cancer genesis and progression and disparities in treatment responses [32,33]. SNPs may potentially cause the lncRNA to behave abnormally, leading to dysregulation of downstream signaling cascades and target gene expression [34,35].

In the present study, we found that *PUNISHER* rs12318065 AA and AC genotype carriers have a considerably higher risk of CC. The A allele was more common in cancer versus non-cancerous tissues and was considered a risk allele for CC under all genetic models. Also, the A/A and A/C genotypes were associated with a greater risk of distant metastases and mortality. Furthermore, nearly 63.8% of the cancer tissues showed a tendency for C>A shift, and the frequency of C>A somatic mutation was shown to be higher in the adenocarcinoma subtype. Additionally, patients harboring C>A somatic mutation were more likely to relapse. To our knowledge, there were no studies that uncovered the relation between *PUNISHER* rs12318065 and CC risk and/or outcome.

Accumulating evidence in the era of lncRNAs genetic variants, notably SNPs, has recently revealed that SNPs in lncRNAs are linked to an increased risk of colon/colorectal cancers. For example, Li et al. [31] reported that the lncRNA “Colorectal Cancer Associated Transcript 1 (*CCAT1*)” rs67085638 C>T was associated with an increased risk of CC, and the rs7013433 A>T was correlated to an advanced stage of CRC in Chinese population. Also, Cao et al. [36] found that the AA genotype of the lncRNA maternally expressed gene 3 (*MEG3*) rs7158663 was significantly increased the CRC risk, in particular, in those over 60 years and with a positive family history of cancer. Another study has demonstrated that the HOXA transcript at the distal tip (*HOTTIP*) rs3807598 (GG vs. CC) and rs2067087 (CC vs. GG) increased the CRC risk by 1.57- and 1.70-fold, respectively, while the rs17501292 variant was associated with improvement in OS of CRC patients with ulcerative/invasive tumors [37]. The metastasis-associated lung adenocarcinoma transcript 1 (*MALAT1*) rs664589 G allele was thought to change MALAT1’s binding to miR-194-5p, resulting in increased gene expression and accelerated CRC growth and metastasis [38]. Similarly, the *GAS5* gene promoter rs55829688 variant was implicated in changing the gene expression via influencing the binding affinity of the transcriptional factor YY1 to the promoter region [39], and the prostate cancer-associated transcript 1 (*PCAT1*) rs2632159 may influence CRC risk by altering EBF, LUN-1, and TCF12 binding, thereby up-regulating PCAT1 expression and hence potentiate its carcinogenic role [40].

Currently, stratified analysis by patients’ characteristics reveals an association between the *PUNISHER* rs12318065 variant and the rate of C>A somatic mutation with decreased OS and DFS. Our data also showed that tumors harboring this type of transversion were a significant independent predictor of overall survival with the presence of distant metastasis. In an attempt to potentially predict the impact of the studied variant on disease risk and/or outcome, we run the “HaploReg v4.1” (https://pubs.broadinstitute.org/mammals/haploreg/haploreg.php) (last accessed on February 2022), which is an updated and validated bioinformatics tool specified for exploring annotations of the non-coding genome variants, based on the 1000 Genomes Project, and predicting the effect of variants on the regulatory motifs and gene expression based on the expression quantitative trait locus (eQTL) studies [41]. Interestingly, the obtained results showed that the *PUNISHER* rs12318065 A variant could influence binding with the transcriptional factors: POL2, STAT1, and ZNF263, which are linked to carcinogenesis [42–46], also it could alter regulatory motifs, including *mrg1, HOXa9_1*, and *Sin3Ak-20_disc6* some of which have been confirmed to be associated with colon cancer carcinogenesis and metastasis [47]. We further evaluated, in silico, the potential function of other SNPs that were in high (*r*^2^ > 0.80) linkage disequilibrium (LD) with rs12318065 and found that some of these polymorphisms were in regulatory regions, including the promoter, the enhancer, and DNase hypersensitivity sites (Table 5). These results could support the potential association of the studied variant with dysregulated gene expression and function that warrant further future functional studies to confirm these speculations.

**Table 5:** Impact and linkage disequilibrium (LD) of the studied rs12318065 polymorphism on chromosome 12 with other variants (*r*^2^ ≥ 0.8) on the same chromosome

pos (hg38)	LD (*r*²)	LD (*D*')	Variant	Ref	Alt	AFR freq	AMR freq	ASN freq	EUR freq	eQTL results	Motifs changed
57670654	0.94	0.98	rs12819172	A	G	0.11	0.22	0.12	0.13		4 altered motifs
57671601	0.92	0.96	rs10876996	G	A	0.12	0.22	0.11	0.13		4 altered motifs
57672194	0.93	0.98	rs12832574	G	A	0.12	0.22	0.12	0.13		Crx, Pitx2
57677732	0.94	0.98	rs12831104	C	G	0.12	0.22	0.12	0.13		SZF1-1
57678579	0.88	0.98	rs12813558	A	C	0.12	0.22	0.12	0.14		EWSR1-FLI1
57681949	0.94	0.98	rs11172299	T	G	0.12	0.22	0.12	0.13		
57691490	0.9	0.96	rs11172302	G	A	0.12	0.22	0.12	0.12		4 altered motifs
57693089	0.94	0.98	rs34854770	C	T	0.12	0.22	0.12	0.13		4 altered motifs
57704963	0.94	0.98	rs11172305	C	T	0.03	0.21	0.12	0.13		PPAR, Pax-1, RORalpha1
57714736	0.94	0.98	rs12424011	C	T	0.02	0.21	0.12	0.13		8 altered motifs
57718654	0.97	0.99	rs2239891	C	A	0.06	0.21	0.12	0.13		NRSF, Nkx2, Pitx2
57723186	0.97	0.99	rs11172310	T	A	0.02	0.21	0.12	0.13		
57725064	0.99	1	rs4760169	T	C	0.05	0.22	0.12	0.13	POL2	6 altered motifs
57726493	1	1	rs12318065	C	A	0.02	0.21	0.13	0.13	**POL2, STAT1, ZNF263**	**Mrg1:Hoxa9, Sin3Ak-20**
57729039	0.99	1	rs12296750	G	A	0.02	0.21	0.11	0.13		
57729328	0.98	0.99	rs11172314	A	G	0.02	0.21	0.11	0.13		18 altered motifs
57731655	0.92	0.96	rs12307841	T	C	0.02	0.21	0.11	0.13		CDP, Cart1, STAT
57733429	0.92	0.96	rs3893002	G	A	0.02	0.21	0.12	0.13	CFOS	4 altered motifs
57734184	0.92	0.96	rs12422249	G	A	0.02	0.21	0.12	0.13		8 altered motifs

Abbreviations: AFR, African; Alt, alternative allele; AMR, American; ASN, Asian; eQTL, expression quantitative trait locus; freq, frequency; EUR, European; hg38, human genome release number 38; LD, linkage disequilibrium; POL2, DNA polymerase epsilon catalytic subunit A; pos, position; Ref, reference allele; STAT1, signal transducer and activator of transcription 1; ZNF263, zinc finger family protein 263. The red labeled variant is the studied polymorphism in this study.

Data source: HaploReg v 4.1. (https://pubs.broadinstitute.org/mammals/haploreg/haploreg.php) (last accessed February 2022).

PUNISHER dysregulation has been associated with several cancers, including the CC, through various mechanisms and molecular pathways [18,48–50]. It was implicated in regulating fibroblast growth factor receptor 1 (FGFR1) expression in CRC by sponging microRNA-497, and its up-regulation was associated with poor patient survival [10]. Other studies have suggested that PUNISHER and LINC-PINT may create a negative feedback regulation loop in colon cancer [51]. Furthermore, PUNISHER could induce endothelial–mesenchymal transition (EMT) and increase CRC cell proliferation, motility, and invasion via targeting the miR-4,668-3p/SRSF1 axis [52]. It also could raise the Cofilin-1 (CFL1) expression that mediates EMT, cell migration, and invasion in CRC via competitively binding to miR-182-5p [52].

The advantage of our study lies in the relatively large sample size, and we are the first, up to our knowledge, to report a significant association between the *PUNISHER* rs12318065 variant and colon cancer risk and poor prognosis. However, the present study lacks the mechanistic and functional works that uncover the specific role of the studied variant in CC. Further studies are warranted to study the impact of this variant on gene expression level and to explore the potential association of this polymorphism with chemoresistance either in CC or other cancer types. More research into novel lncRNA-based genetic biomarkers for predicting CC susceptibility and/or clinical prognosis is recommended.

## Conclusion

In summary, the present study found that the *PUNISHER* rs12318065 C>A transversion is associated with increased colon cancer risk and poor prognostic indicators in terms of short survival time and tumor relapse.

## Data Availability

All supporting data are included within the main article.

## Abbreviations

CFL1: Cofilin-1
CI: confidence interval
EMT: endothelial–mesenchymal transition
eQTL: expression quantitative trait locus
FGFR1: fibroblast growth factor receptor 1
OR: odds ratio
SNP: single-nucleotide polymorphism

## References

[1] Xi Y. and Xu P. (2021) Global colorectal cancer burden in 2020 and projections to 2040. Transl. Oncol. 14, 101174 10.1016/j.tranon.2021.101174 34243011 PMC8273208

[2] Keum N. and Giovannucci E. (2019) Global burden of colorectal cancer: emerging trends, risk factors and prevention strategies. Nat. Rev. Gastroenterol. Hepatol. 16, 713–732 10.1038/s41575-019-0189-8 31455888

[3] Hull R., Francies F.Z., Oyomno M. and Dlamini Z. (2020) Colorectal cancer genetics, incidence and risk factors: in search for targeted therapies. Cancer Manag. Res. 12, 9869–9882 10.2147/CMAR.S251223 33116845 PMC7553623

[4] Xie Y.H., Chen Y.X. and Fang J.Y. (2020) Comprehensive review of targeted therapy for colorectal cancer. Signal Transduct. Target Ther. 5, 22 10.1038/s41392-020-0116-z 32296018 PMC7082344

[5] Malki A., ElRuz R.A., Gupta I., Allouch A., Vranic S. and Al Moustafa A.E. (2020) Molecular mechanisms of colon cancer progression and metastasis: recent insights and advancements. Int. J. Mol. Sci. 22, 10.3390/ijms22010130 33374459 PMC7794761

[6] Chen J., Liu S. and Hu X. (2018) Long non-coding RNAs: crucial regulators of gastrointestinal cancer cell proliferation. Cell Death Discov. 4, 50 10.1038/s41420-018-0051-8 PMC5919979 29736267

[7] Jiang M.C., Ni J.J., Cui W.Y., Wang B.Y. and Zhuo W. (2019) Emerging roles of lncRNA in cancer and therapeutic opportunities. Am. J. Cancer Res. 9, 1354–1366 31392074 PMC6682721

[8] Abushouk A.I., Kattan S.W., Ahmedah H.T. et al. (2021) Expression of oncolong noncoding RNA taurine-upregulated gene-1 in colon cancer: a clinical study supported by in silico analysis. https://www.cancerjournal.net/preprintarticle.asp?id=324167 10.4103/jcrt.JCRT_484_20 36510991

[9] Chen J., Wang Y., Wang C., Hu J.F. and Li W. (2020) LncRNA functions as a new emerging epigenetic factor in determining the fate of stem cells. Front. Genet. 11, 277 10.3389/fgene.2020.00277 32296461 PMC7137347

[10] Hong S., Yan Z., Song Y., Bi M. and Li S. (2020) LncRNA AGAP2-AS1 augments cell viability and mobility, and confers gemcitabine resistance by inhibiting miR-497 in colorectal cancer. Aging (Albany NY) 12, 5183–5194 10.18632/aging.102940 32202509 PMC7138564

[11] Li H., Guo S., Zhang M., Li L., Wang F. and Song B. (2020) Long non-coding RNA AGAP2-AS1 accelerates cell proliferation, migration, invasion and the EMT process in colorectal cancer via regulating the miR-4,668-3p/SRSF1 axis. J. Gene Med. 22, e3250 10.1002/jgm.3250 32639657

[12] Li W., Sun M., Zang C. et al. (2016) Upregulated long non-coding RNA AGAP2-AS1 represses LATS2 and KLF2 expression through interacting with EZH2 and LSD1 in non-small-cell lung cancer cells. Cell Death Dis. 7, e2225 10.1038/cddis.2016.126 27195672 PMC4917662

[13] Qi F., Liu X., Wu H. et al. (2017) Long noncoding AGAP2-AS1 is activated by SP1 and promotes cell proliferation and invasion in gastric cancer. J. Hematol. Oncol. 10, 48 10.1186/s13045-017-0420-4 28209205 PMC5314629

[14] Luo W., Li X., Song Z., Zhu X. and Zhao S. (2019) Long non-coding RNA AGAP2-AS1 exerts oncogenic properties in glioblastoma by epigenetically silencing TFPI2 through EZH2 and LSD1. Aging (Albany NY) 11, 3811–3823 10.18632/aging.102018 31186379 PMC6594811

[15] Zheng Z., Chen M., Xing P., Yan X. and Xie B. (2019) Increased expression of exosomal AGAP2-AS1 (AGAP2 Antisense RNA 1) in breast cancer cells inhibits trastuzumab-induced cell cytotoxicity. Med. Sci. Monit. 25, 2211–2220 10.12659/MSM.915419 30910994 PMC6446658

[16] Tian Y., Zheng Y. and Dong X. (2019) AGAP2-AGAP1 serves as an oncogenic lncRNA and prognostic biomarker in glioblastoma multiforme. J. Cell. Biochem. 120, 9056–9062 10.1002/jcb.28180 30525219

[17] Chen J., Peng X. and Dai Y. (2019) The long non-coding RNA (lncRNA) AGAP2-AS1 is upregulated in ovarian carcinoma and negatively regulates lncRNA MEG3. Med. Sci. Monit. 25, 4699–4704 10.12659/MSM.914766 31233485 PMC6604673

[18] Liu Z., Wang Y., Wang L. et al. (2019) Long non-coding RNA AGAP2-AS1, functioning as a competitive endogenous RNA, upregulates ANXA11 expression by sponging miR-16-5p and promotes proliferation and metastasis in hepatocellular carcinoma. J. Exp. Clin. Cancer Res. 38, 194 10.1186/s13046-019-1188-x 31088485 PMC6518827

[19] Hui B., Ji H., Xu Y. et al. (2019) RREB1-induced upregulation of the lncRNA AGAP2-AS1 regulates the proliferation and migration of pancreatic cancer partly through suppressing ANKRD1 and ANGPTL4. Cell Death Dis. 10, 207 10.1038/s41419-019-1384-9 30814490 PMC6393474

[20] Alidoust M., Hamzehzadeh L., Rivandi M. and Pasdar A. (2018) Polymorphisms in non-coding RNAs and risk of colorectal cancer: a systematic review and meta-analysis. Crit. Rev. Oncol. Hematol. 132, 100–110 10.1016/j.critrevonc.2018.09.003 30447914

[21] Xue Y., Gu D., Ma G. et al. (2015) Genetic variants in lncRNA HOTAIR are associated with risk of colorectal cancer. Mutagenesis 30, 303–310 10.1093/mutage/geu076 25432874

[22] Zheng Y., Yang C., Tong S. et al. (2017) Genetic variation of long non-coding RNA TINCR contribute to the susceptibility and progression of colorectal cancer. Oncotarget 8, 33536–33543 10.18632/oncotarget.16538 28418933 PMC5464888

[23] Zhu Z., Xue Y., Fu W. et al. (2016) Functional indel polymorphism within lncRNA GAS5 and colorectal carcinoma risk. Int. J. Clin. Exp. Pathol. 9, 11767–11773

[24] Li S., Hua Y., Jin J. et al. (2016) Association of genetic variants in lncRNA H19 with risk of colorectal cancer in a Chinese population. Oncotarget 7, 25470–25477 10.18632/oncotarget.8330 27027436 PMC5041918

[25] Amin M.B., Greene F.L., Edge S.B. et al. (2017) The eighth edition AJCC cancer staging manual: continuing to build a bridge from a population-based to a more “personalized” approach to cancer staging. CA Cancer J. Clin. 67, 93–99 10.3322/caac.21388 28094848

[26] Rashid F.A., Tabassum S., Khan M.S. et al. (2021) VE1 immunohistochemistry is an adjunct tool for detection of BRAF. J. Clin. Lab. Anal. 35, e23628 10.1002/jcla.23628 33305405 PMC7891529

[27] Kattan S.W., Hobani Y.H., Abubakr Babteen N. et al. (2022) Association of B-cell lymphoma 2/microRNA-497 gene expression ratio score with metastasis in patients with colorectal cancer: A propensity-matched cohort analysis. J. Clin. Lab. Anal. 36, e24227 10.1002/jcla.24227 34994989 PMC8841134

[28] Kong X., Yang S., Liu C. et al. (2020) Relationship between MEG3 gene polymorphism and risk of gastric cancer in Chinese population with high incidence of gastric cancer. Biosci. Rep. 40, 10.1042/BSR20200305 PMC7685008 33119060

[29] Fawzy M.S., Ibrahiem A.T., AlSel B.T.A., Alghamdi S.A. and Toraih E.A. (2020) Analysis of microRNA-34a expression profile and rs2666433 variant in colorectal cancer: a pilot study. Sci. Rep. 10, 16940 10.1038/s41598-020-73951-y 33037254 PMC7547073

[30] Sameer A.S. (2013) Colorectal cancer: molecular mutations and polymorphisms. Front. Oncol. 3, 114 10.3389/fonc.2013.00114 23717813 PMC3651991

[31] Li Y., Jing F., Ding Y., He Q., Zhong Y. and Fan C. (2018) Long noncoding RNA CCAT1 polymorphisms are associated with the risk of colorectal cancer. Cancer Genet. 222-223, 13–19 10.1016/j.cancergen.2018.02.003 29666003

[32] Cheetham S.W., Gruhl F., Mattick J.S. and Dinger M.E. (2013) Long noncoding RNAs and the genetics of cancer. Br. J. Cancer 108, 2419–2425 10.1038/bjc.2013.233 23660942 PMC3694235

[33] Ling H., Vincent K., Pichler M. et al. (2015) Junk DNA and the long non-coding RNA twist in cancer genetics. Oncogene 34, 5003–5011 10.1038/onc.2014.456 25619839 PMC4552604

[34] Jiang D., Jin M., Ye D. et al. (2020) Polymorphisms of a novel long non-coding RNA RP11-108K3.2 with colorectal cancer susceptibility and their effects on its expression. Int. J. Biol. Markers 35, 3–9 10.1177/1724600819888512 31789575

[35] Poursheikhani A., Abbaszadegan M.R. and Kerachian M.A. (2021) Mechanisms of long non-coding RNA function in colorectal cancer tumorigenesis. Asia Pac. J. Clin. Oncol. 17, 7–23 10.1111/ajco.13452 32970938

[36] Cao X., Zhuang S., Hu Y. et al. (2016) Associations between polymorphisms of long non-coding RNA MEG3 and risk of colorectal cancer in Chinese. Oncotarget 7, 19054–19059 10.18632/oncotarget.7764 26934323 PMC4951351

[37] Lv Z., Xu Q., Sun L. et al. (2019) Four novel polymorphisms in long non-coding RNA HOTTIP are associated with the risk and prognosis of colorectal cancer. Biosci. Rep. 39, 10.1042/BSR20180573 PMC6504661 30940774

[38] Wu S., Sun H., Wang Y. et al. (2019) MALAT1 rs664589 polymorphism inhibits binding to miR-194-5p, contributing to colorectal cancer risk, growth, and metastasis. Cancer Res. 79, 5432–5441 10.1158/0008-5472.CAN-19-0773 31311811

[39] Wang Y., Wu S., Yang X., Li X. and Chen R. (2019) Association between polymorphism in the promoter region of lncRNA GAS5 and the risk of colorectal cancer. Biosci. Rep. 39, 10.1042/BSR20190091 PMC6465203 30902880

[40] Yang M.L., Huang Z., Wu L.N., Wu R., Ding H.X. and Wang B.G. (2019) lncRNA-. Biosci. Rep. 39 10.1042/BSR20190708 PMC6629943 31253700

[41] Ward L.D. and Kellis M. (2012) HaploReg: a resource for exploring chromatin states, conservation, and regulatory motif alterations within sets of genetically linked variants. Nucleic Acids Res. 40, D930–D934 10.1093/nar/gkr917 22064851 PMC3245002

[42] Mullany L.E., Herrick J.S., Wolff R.K., Stevens J.R., Samowitz W. and Slattery M.L. (2017) Transcription factor-microRNA associations and their impact on colorectal cancer survival. Mol. Carcinog. 56, 2512–2526 10.1002/mc.22698 28667784 PMC5633497

[43] Tanaka A., Zhou Y., Ogawa M. et al. (2020) STAT1 as a potential prognosis marker for poor outcomes of early stage colorectal cancer with microsatellite instability. PLoS ONE 15, e0229252 10.1371/journal.pone.0229252 32275681 PMC7147729

[44] Zhao T., Li Y., Zhang J. and Zhang B. (2020) PD-L1 expression increased by IFN-γ via JAK2-STAT1 signaling and predicts a poor survival in colorectal cancer. Oncol. Lett. 20, 1127–1134 10.3892/ol.2020.11647 32724352 PMC7377091

[45] Cui J., Liu J., Fan L. et al. (2020) A zinc finger family protein, ZNF263, promotes hepatocellular carcinoma resistance to apoptosis via activation of ER stress-dependent autophagy. Transl. Oncol. 13, 100851 10.1016/j.tranon.2020.100851 32898766 PMC7486481

[46] Fang L., Ye T. and An Y. (2021) Circular RNA FOXP1 induced by ZNF263 upregulates U2AF2 expression to accelerate renal cell carcinoma tumorigenesis and warburg effect through Sponging miR-423-5p. J. Immunol. Res. 2021, 8050993 10.1155/2021/8050993 34514002 PMC8433034

[47] Bhatlekar S., Viswanathan V., Fields J.Z. and Boman B.M. (2018) Overexpression of HOXA4 and HOXA9 genes promotes self-renewal and contributes to colon cancer stem cell overpopulation. J. Cell. Physiol. 233, 727–735 10.1002/jcp.25981 28464221

[48] Shen S., Li K., Liu Y. et al. (2020) Silencing lncRNA AGAP2-AS1 upregulates miR-195-5p to repress migration and invasion of EC cells via the decrease of FOSL1 expression. Mol. Ther. Nucleic Acids 20, 331–344 10.1016/j.omtn.2019.12.036 32199129 PMC7082499

[49] Xu C., Shao Y., Liu J. et al. (2021) Long non-coding RNA AGAP2-AS1 promotes proliferation and metastasis in papillary thyroid cancer by miR-628-5p/KLF12 axis. J. Bioenerg. Biomembr. 53, 235–245 10.1007/s10863-021-09879-3 33604734

[50] Zheng Y., Lu S., Xu Y. and Zheng J. (2019) Long non-coding RNA AGAP2-AS1 promotes the proliferation of glioma cells by sponging miR-15a/b-5p to upregulate the expression of HDGF and activating Wnt/β-catenin signaling pathway. Int. J. Biol. Macromol. 128, 521–530 10.1016/j.ijbiomac.2019.01.121 30684575

[51] Ji L., Chen S., Gu L., Wang J. and Zhang X. (2021) LncRNA AGAP2-AS1 promotes cancer cell proliferation, migration and invasion in colon cancer by forming a negative feedback loop with LINC-PINT. Cancer Manag. Res. 13, 2153–2161 10.2147/CMAR.S260371 33688258 PMC7936697

[52] Guo Z., Liu X. and Shao H. (2021) E2F4-induced AGAP2-AS1 up-regulation accelerates the progression of colorectal cancer via miR-182-5p/CFL1 axis. Dig. Liver Dis. 10.1016/j.dld.2021.08.002 34838479

